# Extracellular vesicle-associated DNA: ten years since its discovery in human blood

**DOI:** 10.1038/s41419-024-07003-y

**Published:** 2024-09-12

**Authors:** Thupten Tsering, Amélie Nadeau, Tad Wu, Kyle Dickinson, Julia V. Burnier

**Affiliations:** 1 Cancer Research Program, Research Institute of the McGill University Health Centre, Montreal, QC Canada; 2 Department of Pathology, McGill University, Montreal, QC Canada; 3 Gerald Bronfman Department of Oncology, McGill University, Montreal, QC Canada

**Keywords:** Cell biology, Biomarkers

## Abstract

Extracellular vesicles (EVs) have emerged as key players in intercellular communication, facilitating the transfer of crucial cargo between cells. Liquid biopsy, particularly through the isolation of EVs, has unveiled a rich source of potential biomarkers for health and disease, encompassing proteins and nucleic acids. A milestone in this exploration occurred a decade ago with the identification of extracellular vesicle-associated DNA (EV-DNA) in the bloodstream of a patient diagnosed with pancreatic cancer. Subsequent years have witnessed substantial advancements, deepening our insights into the molecular intricacies of EV-DNA emission, detection, and analysis. Understanding the complexities surrounding the release of EV-DNA and addressing the challenges inherent in EV-DNA research are pivotal steps toward enhancing liquid biopsy-based strategies. These strategies, crucial for the detection and monitoring of various pathological conditions, particularly cancer, rely on a comprehensive understanding of why and how EV-DNA is released. In our review, we aim to provide a thorough summary of a decade’s worth of research on EV-DNA. We will delve into diverse mechanisms of EV-DNA emission, its potential as a biomarker, its functional capabilities, discordant findings in the field, and the hurdles hindering its clinical application. Looking ahead to the next decade, we envision that advancements in EV isolation and detection techniques, coupled with improved standardization and data sharing, will catalyze the development of novel strategies exploiting EV-DNA as both a source of biomarkers and therapeutic targets.

## Facts

- Over the past decade of research, new findings demonstrate molecular mechanisms governing active DNA emission occurring across different cell lines.
- The topology of DNA is dependent on EV subtype and cell and tissue origin.
- The molecular cargo identified in EVs can vary depending on the isolation technique.
- EV-associated bioactive molecules can be used for disease diagnosis, patient monitoring, and evaluation of treatment response, particularly in the context of cancer.

## Open Questions

- Is DNA present inside small EVs or associated with the vesicle surface?
- What diseases (and types of biofluids) are most promising for the application of EV-DNA as a liquid biopsy biomarker?
- What are other molecular mechanisms underlying EV-DNA emission?
- Does EV-DNA, once internalized by recipient cells, have downstream impact on gene expression and cellular behavior?

## Introduction. EV/NVEP heterogeneity and cargo

Extracellular vesicle (EV) is a comprehensive term encompassing a diverse array of particles released by various cell types, all encased in a lipid bilayer membrane and devoid of the ability to replicate [1]. In contrast, a non-vesicular extracellular particle (NVEP) lacks a lipid bilayer membrane, has a size smaller than 50 nm, and its molecular biogenesis remains unknown [2]. EVs exhibit high heterogeneity and can be classified based on their size, composition, and origin [1, 3]. While recent studies have reassessed EV nomenclature and composition [4, 5], the International Society for Extracellular Vesicles (ISEV) recommends adopting a nomenclature based on (1) size (small EVs, medium EVs, and large EVs) or defined density (low density, medium density, and high density), (2) molecular composition (e.g. expression of tetraspanin, Annexin A5), and (3) description of conditions or cell of origin (e.g., hypoxic EVs, large oncosomes, apoptotic bodies) [1, 3]. In this review, we adhere to the terminologies endorsed by the Minimal information for studies of extracellular vesicles (MISEV) 2018 and the MISEV 2023 guidelines or utilize the terms “EVs” or “NVEPs” when definitions are not specified.

EVs and NVEPs encapsulate an array of bioactive molecules crucial for cell-cell communication in both physiological and pathological contexts. Recent comprehensive reviews and studies have highlighted distinctive variations in proteomic, nucleic acids, and lipid content between NVEPs and various EV subpopulations [6–8]. While considerable attention has been directed towards certain bioactive molecules, such as proteins and RNA, the exploration of EV-DNA, including mitochondrial DNA (mtDNA), has remained relatively under-explored despite its discovery in human blood more than a decade ago [9]. Although research in this domain has been limited, there is an increasing recognition of the biological significance of EV-DNA [10]. Emerging evidence underscores its potential as a source of biomarkers and diagnostic candidates in diverse pathological conditions, including cancer [11], tuberculosis [12], kidney injury [13], prenatal diagnosis [14], Parkinson’s disease [15], and inflammatory disorders [16, 17].

In this review, we highlight the putative molecular mechanisms of EV‐DNA emission and summarize the current knowledge on EV-DNA function in human health and disease, including intercellular communication, cancer progression, and immune regulation. Collating a decade’s worth of research, we delve into the potential clinical and diagnostic applications of EV-DNA, emphasizing its role in biomarker discovery and non-invasive disease detection and monitoring. Furthermore, we summarize the most common techniques used to characterize the genomic and epigenetic content of EV-DNA. Lastly, we assess the potential functional roles of EV-DNA and its impact on human health and disease.

## Section 1. Types of EVs associated with DNA

### Small EVs

Small extracellular vesicles (sEVs), including classical exosomes, are released through a complex, endocytosis-related pathway involving the orchestrated activity of multiple proteins [18]. In brief, exosome formation begins with inward budding of the endosomal membrane and incorporation of cytoplasmic contents into intraluminal vesicles. These intraluminal vesicles then mature and become encapsulated in the multivesicular endosomes. Finally, either endosomal sorting complex required transport (ESCRT) proteins or neutral sphingomyelinase (nSMase) enzyme, independent of ESCRT, mediates multivesicular endosomes migration towards the cell membrane. This results in fusion with the cell membrane and the subsequent release of exosomes into the extracellular space [19]. Exosomes possess a lipid bilayer membrane with a density ranging from 1.10 to 1.21 g/ml and a size ranging from 40 to 150 nm [20]. Given their endosomal origin, the membrane composition of classical exosomes differs from that of the plasma membrane, featuring surface markers such as cluster of differentiation proteins 9 (CD9), CD63, CD81, and internal cargos Alix, syntenin1, and tumor susceptibility gene 101 protein (TSG101) [3, 5]. It is worth noting that exosomes can lack classical tetraspanin markers (CD63, CD9, CD81) [21] and their surface marker compositions can vary depending on the secretory host cell type [20].

A decade ago, landmark studies demonstrated that tetraspanin and TSG101-positive sEVs isolated from human blood contained genomic DNA (gDNA) representing the entire genome of the host cell [9, 22, 23] (Table 1). These studies, performed on EVs isolated from cancer patients, demonstrated that sEV-DNA accurately reflected the mutational status of the original tumor cells, underscoring the potential of EV-DNA as a liquid biopsy biomarker for cancer detection and metastasis. Subsequent research, summarized in Supplementary Table 1a, has further supported the potential of EV-DNA as a biomarker for various diseases across different biofluids. The discovery of EV-DNA has prompted critical questions about the molecular mechanisms of DNA packaging into sEVs, the topology and biophysical properties of EV-DNA, and its fate.

**Table 1:** EP subtypes and EP-DNA studies organized by EV size.

Extracellular particles	EV or EP subtypes	Markers	Type of DNA	Size	Isolation	Ref
Non Vesicular Extracellular nanoparticles (NVEPs)	Supermeres	TGFBI HSPA13 ENO1 ENO2	None identified	22–32 nm	Ultracentrifugation (367,000 *g* for 16 h)	[71]
	Exomeres	FASN ACLY	dsDNA with histones	28–50 nm	Ultracentrifugation (167,000 *g* for 16 h) OR Asymmetric-flow field-flow fractionation	[2, 71]
	Chromatimeres	EGFR	dsDNA	?	Size exclusion chromatography (7th fraction)	[54]
Extracellular vesicles (EVs)	Small EVs (exosome-like)	CD63 CD81 CD9 TSG101 Syntenin1	Absent	40–200 nm	Ultracentrifugation (100,000 *g* for 70 min) and density gradient ultracentrifugation (100,000 *g* for 18 h)	[5]
	Shed midbody remnants (sMB-Rs)	MKLP1 RACGAP1	Absent	300–600 nm	Density gradient (100,000 *g* for 18 h)	[72]
	Microvesicles	ARF6 Annexin A1 Flotillin β_1_-integrin	dsDNA	150–1000 nm	Ultracentrifugation (10,000 *g* for 30 min)	[34]
	Migrasomes	TSPAN4 Integrin a5 Integrin β1	mtDNA	500–3000 nm	Ultracentrifugation (20,000 *g* for 30 min) with optiprep density gradient (150,000 *g* for 4 h)	[62]
	Large Oncosomes	ARF6 V-ATPase G1 CK18 Annexin A1	dsDNA Chromatin fragments	1000–10000 nm	Ultracentrifugation (10,000 *g* for 30 min) and iodixanol density gradient (100,000 *g* for 3 h 50 min)	[52]
	Apoptotic EVs	Annexin V	dsDNA Chromatin fragments	100–5000 nm	Ultracentrifugation (1,200 *g* for 30 min)	[5, 56]

Summary table of different extracellular particle subtypes (NVEPs and EVs) associated with nuclear DNA and mitochondrial DNA, organized by size.

*ACLY* ATP citrate lyase, *ARF6* ADP ribosylation factor 6, *CD63* cluster of differentiation proteins 63, *ENO1* enolase 1, *ENO2* enolase 2, *FASN* fatty acid synthase, *EGFR* epidermal growth factor receptor, *HSPA13* heat shock protein family A (Hsp70) member 13, *MKLP1* mitotic kinesin like protein1, *RACGAP1* Rac *GTPase*-activating protein1, *TGFBI* transforming growth factor beta induced.

While it is widely acknowledged that various subtypes of EVs or NVEPs carry DNA cargo, few studies have elucidated the molecular mechanisms of double-stranded (ds)DNA loading into small EVs. For instance, Yokoi et al. [24] and Takahashi et al. [25] have both shown that sEVs play a role in maintaining cellular homeostasis by removing cytoplasmic chromatin and micronuclei content—indicators of chromosomal instability and a hallmark of cancer [26]. Moreover, sEV-DNA emission has been observed as a result of nuclear instability and cytoplasmic chromatin leakage [27, 28]. The majority of EV-DNA studies have focused on cancer, and many promising reports highlight the translational application of EV-DNA as a biomarker. For example, studies have shown that plasma-derived sEVs carry mutant DNA (e.g., *EGFR* T790M, *KRAS* G12D and *TP53* R273H) serving as potential biomarkers of colon, lung, and pancreatic cancers [29–31]. These findings suggest that sEVs subtypes harboring DNA, such as exosomes, could complement existing methods of cancer diagnosis and surveillance.

### Microvesicles

The extrusion of outer-membrane vesicles gives rise to another subset of EVs known as microvesicles (MVs), which typically range from approximately 150 to 1000 nm [5]. Unlike exosomes, MVs are produced through direct budding from the plasma membrane, a process regulated by various proteins, including mucin [32], arrestin domain-containing protein 1-mediated MVs [33], Annexin A1 [5] and small GTPases including ADP-ribosylation factor 6 (ARF6) [34]. MVs have garnered attention in the EV-based liquid biopsy field because of their cargo, which includes oncogenes and oncoproteins. Balaj et al. demonstrated that tumor-derived microvesicles (TMVs) carry retrotransposon elements and amplified oncogene sequences [35].

While the mechanism underlying MV shedding remains under active investigation, recent studies showed that active secretion of TMVs can be regulated via ARF6, RhoA, and Rab22A [7, 36, 37]. Similarly, the ARF6 and cyclic guanosine monophosphate–adenosine monophosphate synthase (cGAS) pathways also seem to play a role in transporting DNA into TMVs [34] and selectively sorting pre-miRNA into TMVs [38]. A significant aspect of MV-associated DNA lies in its abundance. Quantitative data on dsDNA content within isolated TMVs (from cell culture conditioned medium) showed an average of 5.609 × 10^-6 ^pg of DNA/TMV [34]. In comparison, recent calculations indicate that plasma exosomes carry much less, such as ~1.4 × 10^−8^ pg of DNA/exosome (healthy donor) and 1.1 × 10^−8^ pg of DNA/exosome (breast cancer patient) [39]. Indeed, reports suggest that the majority of DNA is localized within MVs [40]. Notably, metastatic cells have been shown to exhibit a higher DNA content per TMV [34], further supporting TMV-DNA as a potential biomarker with applications in clinical settings.

### Apoptotic EVs

Apoptotic EVs (Apo-EVs) are characterized by exposure to phosphatidylserine (PS), and presence of caspase-cleaved proteins such as caspases 3 and 7 including PANX1 (plasma membrane) [41], ROCK1 (cytosol) [42, 43] and PARP1 (nucleus) [44] indicative of their release during apoptosis. Apo-EVs exhibit considerable heterogeneity and can be categorized into small Apo-EVs with a size less than 200 nm [45], medium Apo-EVs between 200 nm-1000 nm, and large Apo-EVs ranging from 1000 nm to 5000 nm [46]. However, it should be noted that the size and presence of PS does not indicate Apo-EVs. Discriminating between exosomes, MVs, and small Apo-EVs can be challenging, given their overlapping size ranges [47]. Recent studies have also shown that tumor-derived exosomes display PS in their outer membrane by a mechanism that is unrelated to dying apoptotic cells [48, 49]. Nevertheless, Apo-EVs are identifiable by positive staining for Annexin V [5] and may harbor distinct gDNA fragments compared to MVs and sEVs [50, 51]. Because these particles are released during cell death, research has delved into the impact of drug treatments on Apo-EV release, revealing that both chemotherapy [52] and antibiotic treatments [53] can induce apoptosis-mediated release of various components, including DNA fragments, histones, and cellular organelles. Notably, pre-treatment with a pan-caspase inhibitor (ZVAD) has been shown to reduce large EV-DNA emissions, underscoring the significance of Apo-EVs as a major source of EV-DNA [54]. Furthermore, the antibiotic fluoroquinolone has been implicated in triggering the emission of chromosomal and mtDNA on the surface of small Apo-EVs [53]. Lastly, Apo-EVs exhibit the capability to be internalized by neighboring cells, leading to changes in recipient cell behavior depending on their cargo [55]. In the context of cancer metastasis, chemotherapy-induced Apo-EVs seem to play a pivotal role in pre-metastatic niche formation, promotion of breast cancer stemness, therapy resistance, and proliferation of surviving tumor cells [55]. In the autoimmune context, Apo-EVs carrying DNA have been shown to induce interferon-alpha (IFN-α) secretion from human plasmacytoid dendritic cells via Toll-like receptor 9 (TLR9) activation [56]. Another study demonstrated that Apo-sEVs carrying sphingosine-1-phosphate/sphingosine-1-phopshate receptors enter macrophages via endocytosis, triggering the release of interleukin-1 beta as well as various inflammatory cytokines and chemokines through nuclear factor-κB pathway activation [45]. These studies highlight the potential importance of Apo-EVs in disease progression and immune activations.

### Large EVs

#### Large oncosomes

Large oncosomes (LOs), specialized EVs released by cancer cells, have garnered attention for their unique role in intercellular communication. These LOs, which have shown to be actively secreted from the plasma membrane of metastatic prostate cancer cells [57], typically range from 1 to 10 µm [58], and differ significantly from classical exosomes and MVs. They are distinctive not only in size but also in their cargo, which includes proteins, lipids, and nucleic acids. Notably, the DNA content within LOs has become a focal point of investigation due to its potential implications for cancer biology and liquid biopsy applications [59]. The release of LOs from cancer cells is a complex process intricately linked to oncogenic transformation [57]. It involves the budding and shedding of membrane-bound vesicles containing diverse molecular cargo into the extracellular space. Multiple proteins are involved in the biogenesis of LOs including RhoA/ROCK activation, Ras superfamily GTPases, and ESCRT [58].

Interestingly, actin nucleating protein diaphanous related formin 3 plays an important role in inhibiting LOs emission, whereas the biogenesis of LOs is dependent on the activation of AKT1 and EGFR pathways [57]. The cargo within LOs is diverse, encompassing oncoproteins such as proteins related to metastasis (e.g., GTP-binding protein ARF6 [60], matrix metalloproteinases [61]), and oncogenes [59]. While the understanding of dsDNA topology in sEVs is limited, studies consistently reveal the presence of dsDNA in larger EVs, LOs being a notable example [57, 59]. The presence of dsDNA in LOs becomes particularly significant when considering the implications for genetic material transfer. Vagner et al.‘s findings highlight that the majority of dsDNA is concentrated in large EVs, such as LOs, with only minimal amounts present in sEVs. Moreover, dsDNA within large EVs is bound to histones and reflects the mutational signature of their cells of origin [59]. This emphasizes the potential of LOs as carriers of genetic information reflective of the underlying tumor biology and opens avenues for exploring their utility in liquid biopsy applications, such as for non-invasive diagnostics, monitoring disease progression, and assessing treatment response.

#### Migrasomes

A recent addition to the expanding landscape of EV subtypes, migrasomes represent a distinctive class of large EVs. Aptly named, these vesicles are secreted during cellular migration, and their composition includes cellular organelles [62]. Migrasomes have been observed across various human cell types as well as in several in vivo models including zebrafish [63] and mice [62]. Beyond their prevalence, studies have unveiled multiple roles for migrasomes, such as maintaining cellular homeostasis [64], modulation of the tumor microenvironment [57], and promotion of embryonic angiogenesis [65]. Proteomic data have shed light on the composition of migrasomes, revealing a predominance of proteins associated with the membrane and cytoskeleton. Notably, a small fraction (2%) of nuclear content has also been detected, underscoring the diversity of their cargo [62]. Recent work by Li Yu and Yang Chen identified specific migrasome markers such as bifunctional heparan sulfate N-deacetylase/N-sulfotransferase 1, phosphatidylinositol glycan anchor biosynthesis class K, carboxypeptidase Q and EGF domain-specific O-linked N-acetylglucosamine transferase [66], distinguishing them from sEV markers and potentially offering a means of subtype differentiation. However, the functional significance of these markers in biogenesis and cargo sorting remains to be elucidated.

Beyond protein markers, mature migrasomes have been shown to carry translationally competent full-length mRNA and damaged mitochondria, expanding the scope of their potential intercellular communication [64]. Interestingly, Antje et al. demonstrated the presence of DNA-interacting proteins, raising intriguing questions about the possible inclusion of dsDNA within migrasomes, although this aspect is yet to be conclusively determined [67].

It’s crucial to highlight that migrasomes, although promising, are still in the early stages of exploration. Research on their functional roles is in its infancy, and their implications in various diseases warrant further attention. Several comprehensive reviews on migrasomes have been published [68, 69], providing a foundation for future investigation.

### Non-vesicular extracellular nanoparticles

Recent advancements in isolation techniques have facilitated the purification of non-vesicular nanoparticles or extracellular nanoparticles called exomeres [2] and supermeres [70–72] (the supernatant of exomeres) from the complex mixture of the cellular secretome [73]. This progress adds a layer of complexity to the already heterogeneous landscape of EVs. These NVEPs lack a lipid bilayer membrane and are smaller than sEVs ( < 50 nm). Exomeres harbor unique bioactive molecules, including glycolytic enzymes, and are enriched in fatty acid synthase, ATP citrate lyase, β-galactoside α2,6-sialyltransferase 1, EGFR ligand, amphiregulin proteins, and nucleic acids [74]. This distinctive composition implicates exomeres in various pathological conditions, making them potential biomarkers for various diseases. For instance, studies have shown that exomeres containing amphiregulin enhance the growth of colonic tumor organoids, while those carrying β-galactoside α2,6-sialyltransferase 1 mediate tumor metastatic behavior [74]. Despite these findings, additional functional studies are required to delineate the various roles of NVEPs in conditions such as cancer, neurodegenerative diseases, and cardiovascular diseases. The Lyden group observed that exomeres carry dsDNA fragments exhibiting a unique size range, spanning from 100 bp to 10 kbp. This range significantly differed from the dsDNA fragment sizes observed in both sEVs and large EVs, highlighting variations in the biogenesis mechanisms and structural capacities of distinct particle types [2].

On the other hand, supermeres lack exosome markers (e.g., CD9 and syntenin1) and consist of abundant extracellular RNA (e.g., miRNA-1246), while the presence of DNA has not yet been reported. Supermeres are enriched in metabolic enzymes (enolase1, enolase2, lactate dehydrogenase-A) as well as proteins (transforming growth factor beta-induced, heat shock protein HSPA13) [71]. Another class of NVEPs, known as chromatimeres, were identified to carry EGFR and DNase-resistant chromatin, revealed through nano-flow cytometry coupled with structured illumination microscopy [54]. While the biogenesis of exomeres and supermeres is not fully understood, the presence of a highly enriched retromer complex (VPS35, VPS29, and VPS26) suggests that distinct endosomal export pathways are involved in cargo recruitment and sorting [71].

## Section 2. Molecular mechanisms involved in EV-DNA emission

**Fig. 1:**
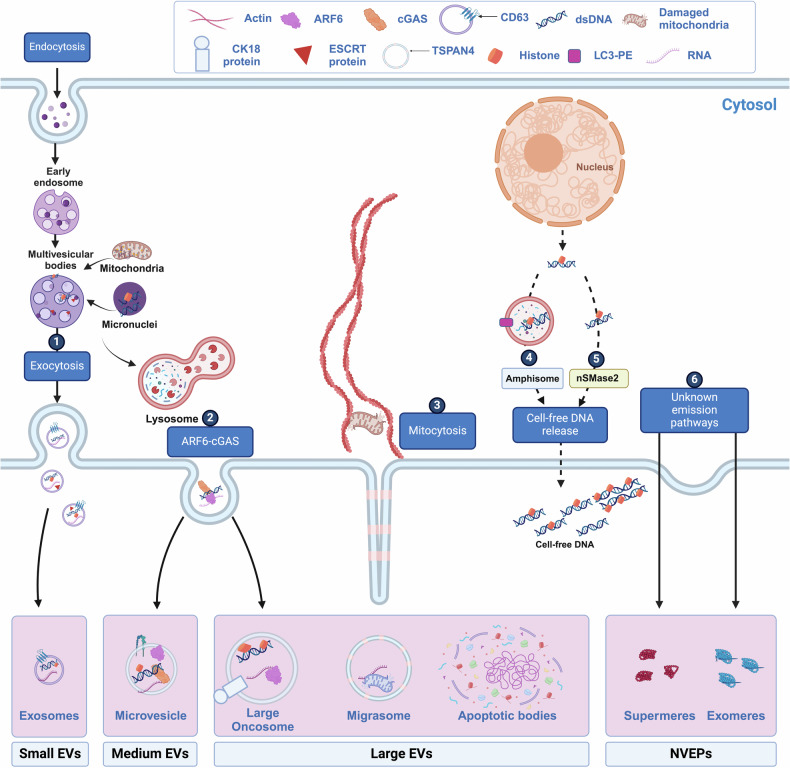
A schematic illustration of extracellular particle biogenesis and possible mechanisms of Extracellular Particles-DNA emission. Extracellular Particles-DNA emission is summarized in two pathways: passive and active secretion. Passive secretion occurs during apoptosis and necrosis, whereas active emission is either associated with sEVs or independent of sEV biogenesis. 1) Cytoplasmic chromatin fragments are shuttled into sEVs via the ESCRT dependent pathway or 2) TMVs via the ARF6-cGAS pathway, subsequently emitted out of a cell. 3) Migrasome carrying mtDNA can be secreted during cellular migration (Mitocytosis). cfDNA can also be secreted via the 4) amphisome and 5) nSMase2 pathways or as 6) NVEP such as exomeres and chromatimeres. Created using Biorender.com.

The current understanding of the molecular biogenesis of EV-DNA and its transport between cells, including the potential involvement of different EV subtypes, remains limited. Gaining insight into the mechanisms governing EV-DNA emission is crucial for unraveling its biological and pathological roles and facilitating its translation into clinical applications. EV-DNA is thought to have diverse cellular sources, including nuclear DNA, mtDNA, and cytoplasmic chromatin fragments [17, 27]. The packaging of EV-DNA might arise from variations in the biogenesis of distinct EV subpopulations including their cellular compartments of origin [2]. In addition, the association between DNA and EVs could be influenced by various cellular processes, such as apoptosis, necrosis, autophagy, migration, and active secretion, could contribute to EV-DNA release into the extracellular space (Fig. 1). Understanding these intricate processes is pivotal to comprehending the broader implications of EV-DNA in both normal biology and pathological conditions.

### Passive secretion of EV-DNA

Apoptosis represents a form of programmed cell death involving a cascade of molecular events that culminate in the elimination of damaged cells, thereby maintaining the overall cell population in tissues. In contrast, necrosis is an unprogrammed form of cell death triggered by external stimuli such as heat, irradiation, mechanical stress or freezing and thawing. A number of studies and reviews have demonstrated that both prokaryotic and eukaryotic cell death processes serve as mechanisms through which cytoplasmic DNA or nuclear components are released into the extracellular environment [46, 75].

Under (patho)physiological conditions, such as cancer, both constitutive and therapy-induced apoptosis may contribute significantly to the emission of EV-DNA and cell-free (cf)DNA [76]. For instance, elevated levels of EV-DNA and cfDNA in the extracellular environment can originate from cancer cells exposed to various apoptosis-inducing agents, including chemotherapeutic drugs like staurosporine [77], camptothecin [52], topotecan, [78] irinotecan, and cisplatin [79], which are widely employed in clinical oncology. Interestingly, Apo-EV DNA exhibits a distinctive laddering pattern in gel electrophoresis, reminiscent of mononucleosome sizes (160 bp-180 bp), possibly due to DNA cleavage by endonuclease enzymes. Conversely, the presence of longer EV-DNA fragments, >10 kbp, is typically indicative of nonspecific degradation associated with necrosis [45, 77]. This nuanced understanding of the distinct patterns in EV-DNA fragments sheds light on the underlying mechanisms and origins, providing valuable insights into the nature of cell death in various physiological and pathological contexts.

Moreover, research has revealed that treatments targeting EGFR, including canertinib and dacomitinib, lead to a significant increase in EV-DNA release from cancer cells [80]. Pre-treatment with a ZVAD has been shown to reduce EV-DNA emission, highlighting the role of the caspase-dependent apoptotic pathway in EV-DNA release [54]. Remarkably, DNA within EVs appears to be protected from DNase I treatment, suggesting that DNA fragments are enclosed within the EV lumen, rather than existing as free nucleosomes [80]. While the exact mechanisms governing EV-DNA release during drug treatments remain elusive, structured illumination microscopy has revealed that DNA is more enriched in EGFR-positive EVs compared to CD63-positive EVs. This suggests that EVs generated following drug treatment may not adhere to classical exosome pathways and are likely of plasma membrane origin [54]. Notably, cells treated with apoptosis-inducing agents, such as staurosporine [76], camptothecin [77], or UV [81], release Apo-EVs containing nucleotides within their lumens as well as externally on the vesicle surface [52]. While apoptosis is widely accepted as one of the mechanisms for the release of intracellular components and nuclear chromatins into the extracellular environment, a pertinent question arises: How do cancer cells, characterized by high chromosomal instability and tumor-promoting mutations that suppress apoptosis, manage to shed dsDNA into the extracellular milieu? This has prompted numerous researchers to explore an alternative mechanism utilized by cancer cells, which involves the active release of DNA rather than through cell lysis [5, 82].

### Active secretion of EV-DNA

The past decade has witnessed a profound exploration of active EV-DNA secretion, a phenomenon that challenges traditional notions of how genetic material is released from cells, particularly in the context of cancer. This resulted in several studies demonstrating potential roles of various proteins involved in EV-DNA emission (Table 2). These studies have been pivotal in shedding light on the active release of DNA from live cells, especially providing valuable insights into the early stages of cancer development. In contrast to apoptosis, which is characteristic of later stages of cancer, active EV-DNA emission offers a unique perspective on how cells maintain cellular homeostasis by removing harmful cytoplasmic DNA, a process which becomes dysregulated during cancer [25, 83]. The DNA can be actively released through encapsulation within membrane vesicles as well as through active release of cfDNA and NVEPs (exomeres) independent of vesicles.

**Table 2:** Molecular mechanisms of EV-DNA emission listed in chronological order of evidence.

Cell type	EV subtypes	EV isolation	Biogenesis mechanism	EV-DNA	Ref
*Streptococcus mutans* (Bacteria)	MVs	Ultracentrifugation	Sortase A	Yes	[90]
Mouse embryonic fibroblasts, Listeria monocytogenes	EVs	Ultracentrifugation	Cyclic guanosine monophosphate–adenosine monophosphate synthase (cGAS) and stimulator of interferon genes (STING) pathway	Yes	[91]
Colon adenocarcinoma (DKO-1) Glioblastoma (Gli36)	sEVs	High resolution density gradient (120,000 *g* for 15 h)	Autophagy and multivesicular endosome dependent	Absent in sEVs	[5]
Ovarian carcinoma (OVCAR-5)	sEVs	40,000 rpm for 120 min	Micronuclei content loaded into CD63-positive exosomes	Yes	[24]
Fibroblast (L929)	Migrasomes	20,000 *g* for 30 min	Migration/Mytocytosis	Yes, EV-mtDNA mutant copies	[64]
Melanoma cell (Lox)	TMVs	10,000 *g* for 30 min	The small GTP-binding protein ARF6 and cGAS pathway	Yes	[34]
Colorectal carcinoma (HCT116)	EVs	Ultracentrifugation	Neutral sphingomyelinase 2 (nSMase2)	Absent in sEVs	[82]

In vitro studies on the molecular mechanisms of EV-DNA emission involving both bacteria and mammalian cells.

DNA in eukaryotic organisms is typically confined to the nuclei or within the mitochondria of cells. However, two seminal studies in 2014 led by Thakur et al. and Kahlert et al. revealed a ground-breaking phenomenon: prostate and melanoma cancer cells release sEVs carrying gDNA representing the entire host genome into the extracellular space [23, 84]. This unexpected discovery marked a significant departure from the conventional understanding of DNA localization within cells. Subsequent research corroborated and expanded upon these initial findings, including studies demonstrating that oncogenic transformation results in the release of sEVs that carry mutant dsDNA [22, 27].

Despite these initial discoveries, further studies in this area ceased for a long time. However, in 2017, Takahashi et al. reignited interest by reporting that sEVs play a crucial role in preserving cellular homeostasis by removing cytoplasmic dsDNA during ectopic expression of oncogenic mutant *HRAS* in fibroblasts. Furthermore, inhibition of sEVs resulted in the DNA damage response in fibroblasts, triggering apoptosis and senescence-like irreversible cell-cycle arrest [25].

However, in 2019, the notion of sEV-associated DNA was challenged. Jeppesen et al. [5] reassessed exosome composition, specifically in DKO-1 and Gli36 cells. Their findings revealed an alternative mechanism wherein these cells employ autophagy proteins to release cytoplasmic chromatin into the extracellular space, independently of sEVs. Later, the same group demonstrated that DNA is associated with NVEPs [74], a finding consistent with previous observations regarding exomeres [2]. These revelations challenge the traditional understanding of sEVs as the carriers of extracellular DNA, highlighting the complexity and diversity of mechanisms involved in the release of genomic material into the extracellular milieu.

In contrast, various studies have shown that dysregulation of the nuclear membrane (emerin [85] lamins) [28] lead to nuclear blebbing [86], formation of cytosolic chromatin fragments (micronuclei), and shedding of EVs containing chromatin fragments [27]. Building on this, Yokoi et al. in 2019 reported a potential source of gDNA in sEVs through micronuclei in the context of ovarian cancer cells [24]. In parallel, Clancy et al. demonstrated that active trafficking mechanisms of dsDNA within TMVs [34] is dependent on the nucleotide-binding site of ARF6 and the cytosolic DNA receptor cGAS proteins, but not on amphisomes nor micronuclei components. Another recent study led by Malkin et al. [82] used a DNA-immunoprecipitation method, revealing that the emission of cfDNA is independent of sEVs. The authors demonstrated that cfDNA is not membrane-encapsulated and is susceptible to DNase I enzyme, consistent with previous findings by Jeppesen [5]. Interestingly, Jeppesen reported that amphisomes play a role in dsDNA emission, while Malkin identified nSMase2 as the mediator for the release of accessible nuclear DNA and mtDNA [82]. Nonetheless, Malkin’s observation aligns with those of Subhash et al. which showed activated neutrophil release of nuclear material mediated by nSMase1 during chemotaxis [86].

While the above-mentioned collection of studies indicates major discrepancies in the field, these may be largely attributed to context, such as distinct cell models, as well as technical considerations like the volume of culture-conditioned medium and the techniques used to isolate EVs. Additionally, cellular responses to environmental stress may lead to distinct EV cargo compositions. For instance, cells under stress release EVs with unique DNA content, differing from Apo-EVs [53, 87]. Notably, Lázaro-Ibáñez et al. demonstrated distinct genomic DNA compositions within different subtypes of EVs, including Apo-EVs, microvesicles and exosomes, in prostate cancer cell lines and patient plasma [50]. Beyond gDNA, mitochondria harboring mutant mtDNA is also selectively enriched in large EVs such as migrasomes, a process known as mitocytosis [64]. These studies underscore the selective and cell-dependent packaging of DNA into distinct EV subtypes, which may be also dependent on the specific cellular origin [21], isolation of distinct EV subpopulations based on size/density [88] and cargo [89]. This emphasizes the importance of considering heterogeneous panels of extracellular particles (NVEPs and EVs) and their unique contributions to DNA emission. Taken together, a decade of research into active EV-DNA secretion from live cells has unveiled a new paradigm in our understanding of DNA release mechanisms in eukaryotic cells.

## Section 3. Topology of EV-DNA

While earlier studies suggested the presence of DNA within the lumen of sEVs [22, 90], recent advancements and refined isolation techniques reveal that sEVs from in vitro cell culture [5, 82], plasma [91] and healthy biofluids [92] carry negligible amounts of dsDNA within the lumen. In line with this, studies have shown that sEV-DNA is mostly present on the surface of sEVs isolated from cell cultures [39, 50, 51, 53]. It is important to emphasize that an intricate bio-corona is formed by DNA-binding proteins on the sEV surface, facilitating the interaction between DNA and the sEVs crown, as shown in EVs isolated from urine [13] and plasma [39]. For example, chromosomal DNA has been identified on the surface of EVs, bound by DNA-binding proteins such as XRCC5, XRCC6, and DHX9 [93]. This DNA presence contributes to the negative charge of the vesicles, and to a certain degree may also contribute to the aggregation of EVs [94]. Additionally, EV-bound DNA has been observed to interact with extracellular matrix components such as fibronectin [53]. However, a compelling study suggests that cfDNA in culture is neither carried within the sEV lumen nor associated with the outer EV membrane. Instead, it predominantly exists as accessible mono- and oligo-nucleosome particles that can be degraded by nuclease enzymes [82]. One possible explanation for the apparent discrepancy is that surface-associated DNA on sEVs may be an artifact introduced during ultracentrifugation steps or incomplete enzymatic digestions because of DNA-binding proteins.

The loading capacity of EVs for DNA may be influenced by their size, with larger MVs encapsulating a greater amount of linear and plasmid DNA compared to smaller sEV-like exosomes [95]. Indeed, studies have demonstrated that sEV-like exosomes cannot load large DNA fragments [39, 95, 96], supporting the findings of several over groups [5, 59, 82, 91] that sEV-like exosomes do not carry DNA within their lumen and very little DNA on their surface. Overall, the results suggest that different subsets of EVs have varying potentials for DNA delivery [97], making MVs or LOs a promising option for liquid biopsy-based EV-DNA biomarkers.

## Section 4. Functional role of EV-DNA

**Fig. 2:**
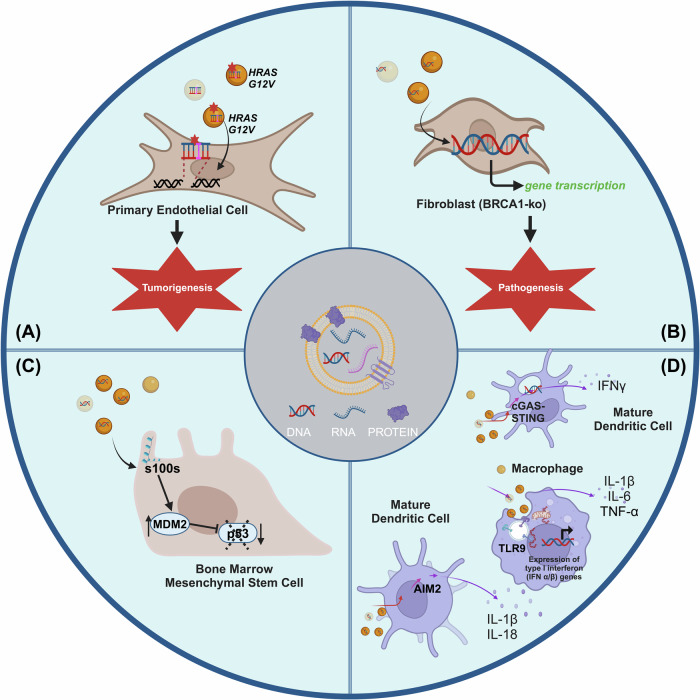
Functional role of EV-DNA: The EV bioactive cargo fuses with the recipient plasma membrane or undergoes endocytosis, ultimately facilitating subsequent signal transduction. **A** Horizontal Gene Transfer: EV-DNA influences oncogene expression in recipient cells with dysregulated tumor suppressor genes. **B** Active transcription of DNA: EV-DNA transferred to recipient cells with a core promoter can be activated depending on the tumor microenvironment and the availability of oncogenic transcription factors. **C** Dysregulation of p53: EV-chromatin and associated proteins (s100s) may dysregulate tumor suppressor protein (p53) modulating the recipient cell’s behavior. **D** Immune modulation: EV-DNA and mtDNA modulate the immune system and release pro-inflammatory cytokines. Created using Biorender.com.

In addition to the roles of EV-associated RNA, proteins, and lipids, EV-DNA (gDNA and mtDNA) may have physiological significance and influence the function of recipient cells. Various studies have indicated that EV-DNA, particularly from cancer cells, can be transferred to recipient cells, potentially resulting in transcription within the target cells and subsequently triggering cellular transformation [34, 98, 99]. Apart from cancer, bacterial MVs have been shown to carry DNA (along with peptidoglycan and RNA) that can activate downstream signaling and function [100]. For example, bacterial MVs carrying DNA can be detected by TLR9 and modulate innate immune responses in the host cells [100]. One study demonstrated that single-cell gram-negative bacteria, non-typeable *Haemophilus influenzae*, actively release DNA into the extracellular environment to support the structural integrity of biofilms, a critical factor in their resistance to treatments for various respiratory tract diseases in humans [101]. In this section, we will focus on studies examining the functional role of EV-DNA, specifically in oncogenic transformation, immune regulation, and disease pathogenesis (Fig. 2).

### Horizontal gene transfer

Unlike conventional vertical gene transfer from parents to offspring, horizontal DNA transfer is a rare occurrence within eukaryotic cells, but it is prevalent among prokaryotic cells. Despite this, investigating the role of EVs in horizontal gene transfer and genometastasis has revealed intriguing findings. Lee et al. demonstrated that EV-DNA carrying *HRAS* mutant copies can be transferred to intestinal epithelial cells, causing a transient alteration in phenotype [102]. This transient effect was attributed to the recipient cells requiring pre-existing alterations in tumor suppressors and genetic instability for permanent transformation. Later, the same group showed that EVs carrying the *HRAS* oncogene can trigger genomic instability and result in micronuclei formation in endothelial cells. Moreover, the recipient cells displayed notable levels of γH2AX phosphorylation and an upregulation of *TP53* expression. These observations are indicative of a robust DNA damage response and cellular stress within the experimental context [103]. These results suggest that DNA damage response and tumor suppressor proteins (p53 or Retinoblastoma) may be involved micronuclei formation in the recipient cells [27] (Fig. 2A**)**. Horizontal transfer of DNA between eukaryotic cells may induce chromosomal disruption crucial for tumor formation [104]. Abdoub et al. expanded on these findings by showing that EV-DNA, mRNA, and miRNA are successfully transferred to single oncosuppressor-mutated fibroblast cells (*BRCA1*-knock out). This transfer not only altered the phenotype of the cells but also induced malignancy in vivo in mice [105]. A possible underlying mechanism behind the induced malignancy may be the decreased expression of cell cycle progression inhibitor CDKN1A and cell death inducer mouse double minute 2 (MDM2), together with increased expression of the oncogenes MYC, HRAS and the antiapoptotic factor BCL2L1 [98]. These findings collectively suggest that nucleic acids associated with colon cancer, transferred via EVs, have the capability to modulate the expression of transcription factors. This modulation ultimately instigates a shift in the fate of BRCA1-KO fibroblasts, mediated through activation of a mesenchymal to an epithelial phenotype transition (MET) and the regulation of cellular growth and apoptosis [98]. Notably, EV-DNA remained functionally active within the recipient cells (Fig. 2B**)**. Another study led by Andreeva et al. suggested that mutant DNA in sEVs alone is not sufficient to induce malignant transformation of recipient cells; rather, it requires a combination with additional cancer-initiating agents [106]. For instance, glioblastoma EVs carrying NANOGP8 DNA promotor sequence and transcriptional regulatory components can mediate transformation from normal or cancer cells to cancer stem cells [107].

Cai et al. demonstrated that *SRY* (sex-determining region Y) DNA in plasma EV was transferred to HUVEC cells, resulting in newly synthesized SRY protein and accelerated atherosclerosis in an in vivo mouse mode [99]. Similarly, Clancy et al. showed that the presence of histones indicates that gDNA within TMVs is available for transcription and gene expression when transferred to recipient cells [34]. This was further confirmed by nano-flow cytometry analysis [78].

It is worth emphasizing that the presence of both DNA and proteins within EVs can impact recipient cells, as demonstrated recently by Ghanam et al. In their study, EVs positive for chromatin and S100 proteins downregulated the expression of some cell cycle genes (p53 and p21) and apoptosis genes (PUMA and BAX), and upregulated MDM2 homolog in bone marrow-derived mesenchymal stem cells. S100 proteins are calcium-binding proteins that interfere with different regulators of cell proliferation and differentiation, including ubiquitination and degradation of p53 [108]. S100 proteins (S100A4 and S100B) bind to the tetramerization domain of p53 and prevent p53-dependent transcription activation [109]. Whereas, MDM2 inhibits p53 acetylation by binding and reducing p300/CREB binding protein acetyltransferase activity or interaction with histone deacetylase 1 (HDAC1) to deacetylate p53, resulting in MDM2‐dependent ubiquitylation and promoting p53 degradation by removing these acetyl groups [110, 111]. Dysregulation of tumor suppressor function may not only have the potential to enhance tumor growth but also facilitate horizontal gene transfer of EV-DNA [112] (Fig. 2C**)**. The capability of eukaryotic cells to engulf EVs carrying genetic material raises the possibility of horizontal gene transfer. However, chromosomes within the nucleus of eukaryotic cells are stably protected through a compartmentalized nuclear membrane. A critical question arises: How is foreign functional DNA integrated and retained within the host’s compartmentalized genome? If foreign functional DNA is retained, is it lost over a prolonged period of time? Moving forward, more studies are needed to understand the mechanism involved in horizontal gene transfer and the exact biological function of DNA sequences in in vitro and in vivo models.

### EV-DNA in inflammation

EVs have been shown to have major impact on immune function in a variety of contexts [113], most notably cancer. Tumor-derived EVs can exert modulatory effects on both the innate and adaptive immune systems [113], and depending on the specific cargo carried on the EVs, the effects can be either immunostimulatory or immunoinhibitory [114]. For example, cancer-derived sEVs have been shown to transfer DNA to dendritic cells [115] and trigger anti-tumor immune responses [116]. Conversely, sEVs from metastatic melanoma, which carry PDL-1 on their surface, inhibit CD8 + T cells, promoting tumor growth [114, 117]. EVs carrying DNA cargo can induce inflammation in recipient cells through three major pathways: absent in melanoma 2 (AIM2), cGAS, and TLR9 (Fig. 2D). Lian et al. demonstrated that chemotherapy induces cytotoxicity in intestinal epithelial cells, resulting in the emission of EV-DNA, which is then uptaken by dendritic cells and macrophages, triggering inflammation via the AIM2 inflammasome [79].

EV-DNA can also stimulate the production of cytokines, particularly type 1 interferon (IFN-I), by innate immune cells. This stimulation involves TLR9 as well as cGAS and depends on the nucleic acid content of the complexes. For example, TLR9 specifically recognizes hypomethylated CpG motifs and induces IFN-I and other inflammatory genes [118]. However, these motifs occur more commonly in bacterial DNA than mammalian DNA because of CpG suppression in mammalian DNA and the lack of cytosine methylation in bacterial DNA [119]. On the other hand, during sterile-inflammation, EVs containing mtDNA rich in CpG dinucleotides can activate TLR9 in macrophages, upregulating pro-inflammatory cytokines [120]. Another study demonstrated that irradiated breast cancer cells trigger the emission of tumor-derived EVs carrying dsDNA, which can be subsequently transferred to dendritic cells, resulting in activation of IFN-I via the cGAS and stimulator of interferon genes (STING) pathway [115]. Similarly, topotecan, an anticancer agent, results in emission of sEVs containing immunostimulatory DNA, thereby activating STING-dependent signaling [116]. Another study reported that plasma-derived EV-DNA induces STING-mediated proinflammatory responses in the context of dermatomyositis [16].

Mechanistically, endogenous DNases are the first line of defense against elevated levels of cfDNA in plasma. As such, defective DNase activity may play an important role in autoimmune diseases such as systemic lupus erythematosus. Notably, in pediatric systemic lupus patients, DNase1L3 was inactivated by neutralizing autoantibodies, leading to the presence of Apo-EVs carrying long polynucleosomal DNA, suggesting a positive correlation between EV-DNA and autoimmune disorders [121].

The activation of the immune system by EV-DNA is not restricted to gDNA alone; EV-mtDNA has also been shown to have an immune-modulatory function [122]. While many studies focus on cancer, Tsilioni et al. demonstrated that serum-derived EVs carrying mtDNA from children with autism spectrum disorder stimulate microglia and induce pro-inflammatory cytokines such as interleukin-1 beta [123]. There are numerous other contexts where EV-mtDNA has been implicated. Interestingly, an in vivo study demonstrated that mice subjected to chronic alcohol consumption, coupled with binge drinking, exhibited an elevation in mtDNA-enriched EVs. These mtDNA-enriched EVs were identified as potential contributors to neutrophilia through TLR9 activation, consequently promoting liver injury [124].

The above-mentioned studies highlight the potential role of EV-DNA in eliciting immunological effects via stimulating TLR and non-TLR nucleic acid sensors, triggering antigen-specific antibody responses and form immune complexes. These processes can drive cytokine production and deposit in tissues, thereby contributing to the development of various autoimmune diseases, such as rheumatoid arthritis, systemic lupus erythematosus [125], and atherosclerotic heart disease [99]. However, despite the potential role of EV-DNA in different pathological conditions, challenges exist in isolating pure EVs and deciphering the specific cargo responsible for phenotypic changes in recipient cells. Indeed, EV cargo is a highly heterogenous mixture of various bioactive molecules. Importantly, current EV isolation techniques purify both EVs and other co-isolated proteins, lipids, and RNA. These contaminants may also contribute to changes in recipient cells, resulting in disease onset and progression. This poses a challenge in pinpointing specific EV cargo responsible for changes in the phenotype and genotype of recipient cells. The MISEV 2023 guidelines recommend rigorous study designs, including dose-dependent response studies and controls, to address these challenges [126]. While the complex cargo of EVs poses difficulties in pinpointing specific influences, studies suggest a potential role for EV-DNA in immunological effects, antigen-specific antibody responses, and autoimmune diseases [127].

## Section 5. Applications of EV-DNA

### EV-DNA as a biomarker in liquid biopsy

Liquid biopsy, the analysis of biological fluids such as blood, saliva, urine, or cerebrospinal fluid, offers a new way to obtain information on both healthy and disease states, with particular significance in cancer research and diagnostics. Due to tumor heterogeneity and evolution, as well as cancer spread to different organs during metastasis, a solid tissue biopsy can fall short in representing cancer’s diversity. To address these challenges, liquid biopsy offers a non-invasive means for the longitudinal sampling of cancer tissue. Within a liquid biopsy, tumor-derived molecules including circulating tumor cells [128] circulating tumor DNA (ctDNA), and tumor-derived EVs can be isolated and characterized. These components play pivotal roles in tumor sampling, facilitating longitudinal monitoring, personalized therapeutic regimens, and screening for therapeutic resistance. The potential applications of EV-DNA for liquid biopsy, along with its functional role and therapeutic implications, are highlighted in Supplementary Table 1a-c. Circulating EVs originate from a comprehensive cell population, therefore holding the potential to reflect the entirety of a heterogeneous tumor [129], and have diagnostic applications. A decade ago, EVs from human plasma were found to contain both gDNA and mtDNA [9]. Since then, it has been well established that tumors abundantly release EVs into the blood, and that cancer patient blood displays elevated levels of EV-associated proteins [130], EV-DNA [23], and EV-RNA [131] compared to normal healthy human plasma/serum. Both ctDNA and EV-DNA can be readily isolated from biofluids of cancer patients, employing less invasive procedures that enable more frequent and repeatable sampling compared to traditional tissue biopsies. (Box 1). However, ctDNA has a limited half-life ranging from minutes to hours [132] and is susceptible to nuclease degradation in circulation [133]. In contrast, the lipidic bilayer membrane of EVs provides protection [134] and stability [135] to EV-associated nucleic acids in biofluids, potentially offering advantages over ctDNA. Given that EVs encapsulate protected cargo that reflect cell-specific pathological processes, they harbor significant potential as circulating biomarkers, offering a promising avenue to enhance current cancer monitoring, prognosis, and therapeutic strategies. This is reflected in number of studies employing droplet digital (dd)PCR and various sequencing methods to detect EV-associated mutant DNA copies [10] (Box 1). In human plasma of patients with prostate cancer, gDNA within EVs has been shown to be packed in a multitude of EV subtypes [50]. Mutations detected in exosomal DNA within regions housing crucial tumor-related genes such as *KRAS* and *TP53* have been identified as potential indicators of primary cancers such as pancreatic cancer [23]. Moreover, EV-associated mutant DNA (*BRAF* V600E) copies can be used as a marker for tumor progression in melanoma patients [136]. In patients with glioma, *IDH1* G395A gDNA sequences were identified in EVs isolated from peripheral blood [137]. Furthermore, *PIK3CA* mutations have been detected in ctDNA and EV-DNA in the plasma of patients with metastatic breast cancer, even in cases where the primary tumors lacked such mutations [138]. A promising study investigated the significance of EV-DNA for colon cancer diagnosis and surveillance using ddPCR. By taking advantage of the most common colon cancer mutations, *KRAS* G12D and G13D, Choi et al. were able to achieve 76.67% sensitivity and 100% specificity using EV-DNA as a biomarker [30]. The fractional abundance of EV-DNA was higher than cfDNA, even when the concentration of EV-DNA was significantly lower than that of cfDNA. The fractional abundance of EV-DNA mutant copies was also shown to positively correlate with the carcinoembryonic antigen levels and overall survival, demonstrating that EV-DNA is a complementary tool to carcinoembryonic antigen measurements widely used in clinical settings.

Indeed, several studies have shown that compared to ctDNA alone, the combination of EV-associated nucleic acids with ctDNA can improve the sensitivity and specificity of cancer detection and disease progression [31, 127, 139]. However, other comparison studies between ctDNA and EV-DNA indicated that ctDNA exhibits higher sensitivity and specificity, particularly in breast cancer [91] and melanoma [140], bringing to light questions on the clinical value of EV-DNA [140, 141]. Given the conflicting data in the field, it is becoming clear that the utility of different liquid biopsy-based tests (e.g., cfDNA *vs*. EV-DNA) may be context-dependent and should be further investigated.

Beyond EVs originating from tumors, circulating EVs derived from immune cells can serve as biomarkers aiding in the prediction of clinical response to chemotherapy. Elevated levels of surface markers PD-1 and CD28 on T cell-/dendritic cell-derived EVs in metastatic melanoma patients have been correlated to improved clinical response to chemotherapeutic drugs like ipilimumab [142]. The versatility of EV-associated bioactive molecules from different etiologies positions them as optimal biomarkers to address the urgent clinical demand for non-invasive liquid biopsy tools across various diseases, particularly cancers.

Despite the considerable advantages offered by EVs, their utilization as a liquid biopsy tool is still in the early phases of development and faces various challenges, including issues related to isolation, purity, yield, and downstream analyses such as detection, sensitivity, specificity, and most importantly, reproducibility among studies. Nevertheless, it is important to consider biofluid selection for EV-DNA biomarker discovery in specific disease contexts. For example, most studies have used plasma [10, 143] and serum [23, 144] for EV-DNA isolation; however, in serous sarcoma (ovarian cancer), ascites sEV-DNA showed more similar copy number variations to tumor tissue than plasma exosomes from the same patient [24]. Another comparative study analyzing urine vs. serum demonstrated that urine EV-DNA performed better than serum EV-DNA in the context of bladder cancer [11]. Similarly, cerebral spinal fluid performed better than plasma in detecting tumor-specific alterations in glioblastoma patients [145]. Furthermore, the challenge of identifying the origin and etiology of EVs hampers the ability to differentiate them by cell type. This difficulty restricts the precise detection of specific EVs within the diverse array of circulating vesicles.

In general, there is a notable scarcity of investigations into EV-derived DNA from human biofluids compared to studies focusing on other components of EV cargo, such as RNA, lipids, and proteins. Currently, there is ongoing exploration of EV-based biomarkers, cell-free therapeutic agents, drug delivery carriers, and cancer vaccines, with various clinical trials registered on the Clinical Trials database (https://clinicaltrials.gov/) yielding diverse outcomes. Recently, the Food and Drug Administration granted a breakthrough device designation to the ExoDx Prostate IntelliScore test, becoming the first exosome-based liquid biopsy test to obtain such a designation. Importantly, the EPI test score demonstrated superior performance compared to the current standard of care in risk stratifying prostate cancer patients [146].

Despite the attractiveness of analyzing EV-DNA from human biofluids for diagnosing and monitoring patients over time, the lack of standardized methods, as well as reproducibility, transparency and data sharing, poses significant challenges. Addressing these issues requires overcoming numerous technical challenges, especially to prevent the co-isolation of vesicles sharing similar physical and molecular properties with EVs. To tackle these challenges, the Rigor and Standardization Subcommittee of the ISEV assembled experts to establish specialized EV Task Forces. For example, a task force now focuses on the specific and unique challenges in blood EV biomarkers, as blood remains the most extensively studied liquid biopsy [147]. In line with this, a recently created Minimal Information for Blood EV research (MIblood-EV) aims to facilitate transparent reporting of plasma and serum preparations [148]. Similarly, the Urine Task Force provides recommendations for more rigorous and reproducible methodologies in urinary EV research to facilitate successful transitions into clinical practice [149].

Crowdsourced repositories are also helping in curating and consolidating publications focused on EV-associated RNA, fostering standardization and progress within the EV community [150]. Until recently, EV-DNA data remained buried in the published literature. To address this, our group developed Extracellular Vesicle Associated DNA database (EV-ADD), a publicly available and free database aimed at enhancing standardization and transparency in the rapidly growing field of EV-DNA research [10].

Box 1 Methodologies to analyze EV-DNA Analysis of EV-DNA involves sampling of different starting material (e.g., culture media, liquid biopsy), followed by extraction and purification of EVs, and finally analysis of EV-DNA. However, the limited presence of tumor-derived EVs within the bulk EV population poses challenges for detecting their content in complex samples such as biofluids. Traditional methods like ELISA and flow cytometry face limitations [97], including the challenge of low concentrations of tumor-derived EVs, resulting in underestimated fractions of EVs containing DNA fragments, necessitating more sensitive tools for clinical settings. According to the EV-ADD, ddPCR and various sequencing methods have been widely used to detect EV-associated mutant DNA copies [10] (Supplementary Figures 1 and 2). Indeed, ddPCR can detect rare mutant copies rapidly, with an additional pre-amplification step helping to detect low copy numbers [13], but requires prior knowledge of hotspot mutations and is limited to known sequences.

In such scenarios, the application of next-generation sequencing (NGS) on EV-DNA offers the distinct advantage, as it can identify unknown single nucleotide polymorphisms as well as detecting all classes of variants (structural variants such as insertions and deletions), which are primarily attributed to the larger DNA fragments carried by EVs [127]. In the realm of EV-DNA studies, Illumina dominates the current NGS landscape, specifically in whole genome sequencing and whole exome sequencing [24, 157, 158]. Beyond genetic studies, epigenetic profiling of EV-DNA, such as methylation analysis, has also been used for tumor classification in glioblastoma, despite low EV-DNA yield [145]. Epigenetic analysis [159, 160] and fragmentomics profiling [161] of cfDNA revealed tissue specific signatures, providing information about health and their tissue of origins [162]. These techniques have proven effective in deciphering the complexities of cfDNA molecular features, may also hold potential for studying the origin of EV-DNA. Looking ahead, analysis of EV-DNA as a liquid biopsy analyte has the potential to personalize treatment decisions, predict cancer outcomes, and identify resistance to therapy, at least in certain cancer types [23, 29, 163].

### EV-DNA therapy

Numerous studies have also highlighted the potential of EV-DNA as a therapeutic strategy, particularly in cancer vaccines [115, 151], where EV-DNA shows promise in priming the immune system to mount defenses against cancer. An encouraging study demonstrated that tumor-derived exosomes containing dsDNA from irradiated cells induced anti-tumor inflammation by inducing IFN-I production from dendritic cells in a STING-dependent manner [115]. In addition, DNA-based vaccines encoding EV-associated ovalbumin antigen have been shown to trigger an anti-tumor immune response, leading to a reduction in tumor progression in vivo [152].

Exogenous DNA can also be packaged into EVs and delivered to target cells, altering their function. For example, an exosome-based nano delivery system was developed to deliver a wild type p53 plasmid to p53-null H1299 cells and p53-knockout mice models, showing no toxicity [153]. Another study demonstrated that large EVs and sEVs derived from a placental explant culture system can be loaded with up to 1000 ng of plasmid, serving as a delivery vehicle for gene therapy [96]. Given the immense heterogeneity of EVs, it remains important to consider their loading capacity, as well as the size and structure of the encapsulated DNA molecules to optimize gene therapy [95]. Moreover, gaining insights into mechanisms of EV-DNA emission and uptake can help us to develop therapeutic strategies aimed at inhibiting the interactions between disease-causing EVs and recipient cells. For instance, autophagy or nSMAse inhibitors have been successfully employed to prevent the EV-mediated transfer of harmful genetic material in several models [27, 82]. This underscores the importance of understanding EV-DNA release mechanisms to exploit them therapeutically. Currently, EV-DNA therapeutics faces several persistent challenges. Firstly, the inherent heterogeneity of cargo within EVs poses challenges for ensuring consistent and efficient delivery of therapeutic payloads. Although electroporation has been widely used to load therapeutic cargo [154–156], it exhibits limited loading capacity for DNA payloads [95], further hindering effective delivery. Moreover, addressing the issue of transient protein expression after DNA transfer in the recipient cells is paramount to achieve sustained therapeutic outcomes [95]. Furthermore, successful clinical trials are needed for validation and eventual clinical implementation of EV-DNA therapeutics. However, thus far, clinical trials have not been successful in showing the clinical utility of EV-DNA. For example, phase 1 clinical trial exoSTAT6 (NCT05375604) was terminated due to company bankruptcy. Moreover, a phase 2 clinical trial (NCT01159288) utilizing dendritic cell-derived EVs (Dex) coupled with tumor antigenic peptides for treating non-small cell lung cancer revealed minimal to no therapeutic benefits, primarily due to low loading efficiency of the therapy. Overcoming these technological challenges is essential for clinical implementation of EV-DNA therapeutics.

## Conclusions and future insights

Over the past decade since the discovery of EV-associated DNA, its pivotal role in both normal physiological and pathological conditions have become evident, including implications in horizontal gene transfer, genetic instability, inflammation, and cancer. However, its discovery has raised important questions surrounding the molecular mechanisms of DNA packaging into EVs. While cell lysis is recognized as a potential contributor to DNA emission into the extracellular space, a growing body of evidence highlights active mechanisms driving DNA emission. This is exemplified by studies revealing the relative abundance of DNA in specific EV subpopulations, indicating a novel form of DNA release from viable cancer cells [59, 74].

However, it is crucial to acknowledge the existing discordance in EV-DNA studies reported in the literature, as highlighted by multiple reviews [6, 17]. Notably, even within the same laboratory and using the same cell line, contradictory findings on the absence or presence of EV-DNA have been reported [5, 74]. Discordant findings within the literature may be partly explained by differences in EV isolation, characterization of cfDNA [82] and EV-DNA detection methods, highlighting the need for standardization, transparency, and data sharing in the field.

In conclusion, our understanding of the cellular trafficking of EV-DNA has advanced significantly over the past decade, with the mapping of pathways and proteins that drive these pathways to some extent characterized. Further studies are needed to characterize the cytoplasmic DNA trafficking pathways. Advancement in our knowledge of EV-DNA biogenesis holds promise for the discovery of novel biomarkers and development of therapeutic strategies, such as inhibiting the interaction between oncogene-loaded EVs and recipient cells. As we enter a new era marked by state-of-the-art EV isolation techniques, as well as international collaborations and task forces, the next decade promises excitement and a better understanding of EV-DNA emission and optimal isolation of EVs. Future work should focus on standardization of biomarker detection as well as improved sensitivity and specificity. Moreover, in the therapy space, efforts should be made to balance EV-DNA therapeutic efficacy and toxicity. Such progress will enhance our ability to fully harness the vast potential of EVs, facilitating their translation from bench to bedside and may provide new insights into their pathogenesis.

## Supplementary information

Supplementary Figures

Supplementary Tables
