# Supplementary material for: Extracellular vesicle-associated DNA: ten years since its discovery in human blood
Source: Cell Death Dis. 2024 Sep 12;15(9):668. doi: 10.1038/s41419-024-07003-y (PMC11393322; doi:10.1038/s41419-024-07003-y)
Supplement: Supplementary file 1 — Supplementary Figures [file 41419_2024_7003_MOESM1_ESM.docx]

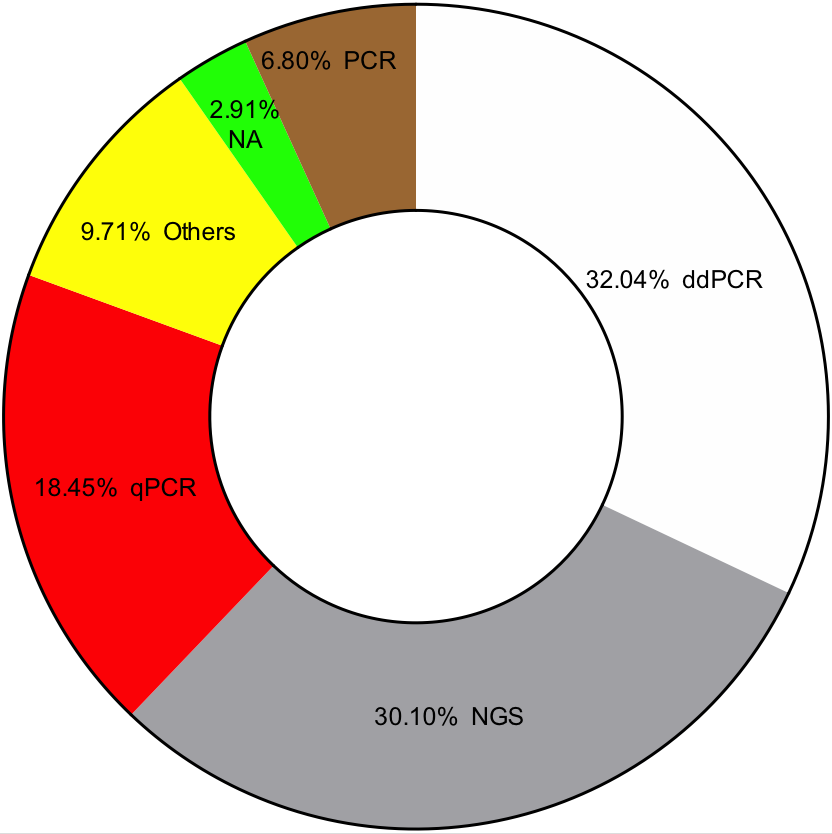


**Supplementary Figure 1**. Techniques used to detect EV-DNA. EV-DNA mutations, copy number variation, and structural variation can be quantified using various techniques such as PCR, qPCR, ddPCR and high throughput NGS. PCR= polymerase chain reaction, qPCR= Quantitative PCR, ddPCR=droplet digital PCR, NGS= next generation sequencing, NA= Not available.

**Supplementary Figure 2**. Techniques used to quantify EV-DNA.
