# Supplementary material for: Extracellular vesicle-associated DNA: ten years since its discovery in human blood
Source: Cell Death Dis. 2024 Sep 12;15(9):668. doi: 10.1038/s41419-024-07003-y (PMC11393322; doi:10.1038/s41419-024-07003-y)
Supplement: Supplementary file 2 — Supplementary Tables [file 41419_2024_7003_MOESM2_ESM.docx]

**Supplementary table 1 a):** EV-DNA Liquid biopsy application, sorted in the alphabetical order of diseases.

| **Disease** | **Results** | **Applications** | **Genes cargo** | **References** |
| --- | --- | --- | --- | --- |
| Acute myeloid leukemia | The acute myeloid leukemia plasma derived EVs carry dsDNA that reflect the mutational status of the cells of origin. | Diagnosis and treatment response | EZH2, ETV6, FLT3-ITD, FLT3-TKD, FLT3-WT, GATA2, KIT, NPM1, NOTCH, NRAS, PHF6, RAD21, WT1, ZRSR2 | Kontopoulou et al 2020 Annals of Hematology |
| Bladder cancer | Multi-omic exosome analysis of bladder cancer patient tissue fluids using the next generation sequencing of somatic DNA mutations, miRNAs, and the global proteome | Biomarker | EGFR | He et al 2022 Communications Biology |
|  | Urine exosome DNA is superior to serum exosome DNA for mutation analysis for bladder cancer. | Biomarker | AHRR, AKAP13, CCL16, CDH1, CTSB, DPH1, ESR2, ETV6, FGF1, FGFR3 GAS7, GDF15, GPI, KRAS, LDLR, MT1B, PCM1, PLG, RNF213, RXRA, TEP1, TP53, VHL | Zhou et al 2021 Molecular Therapy Method & Clinical Development |
| Breast cancer | EV DNA for CNV detection is lower than that of ctDNA. | Liquid biopsy | BRAC1, BRAC2, ERBB2 | Ruhen et al 2020 Biomedicines |
|  | Compare to total cfDNA, EV-DNA does not add value in detecting mutant copies in metastatic breast cancer. | Liquid biopsy | AKT, ESR1, PIK3CA | Tkach et al 2022 Journal of Extracellular Vesicles |
|  | EV-DNA might improve the sensitivity of liquid biopsy | Liquid biopsy | PIK3CA | Nakai et al 2022 Journal of Nippon Medical School |
| Breast lesions | HPV16-DNA as liquid biopsy for breast lession diagnosis. | Diagnosis | HPV16 | Carolis et al 2017 Future Medicine |
| Colon carcinoma | Both cfDNA and EV-DNA can serve as tumor biomarkers | Liquid biopsy | ACVR2A, APC, BRAF, KRAS, PIK3CA, RNF43, TGFBR2, TP53 | Thakur et al 2021 Genes |
| Colorectal cancer | Monitoring of cetuximab-resistant gene mutations in EV DNA | Diagnosis | BRAF, EGFR, KRAS, NRAS, PDFRA, PIK3CA, PTEN | Zhoa et al 2022 Japanese Journal of Gastroenterology and Hepatology |
| Glioblastoma | PD-L1 DNA was present in circulating EVs from glioblastoma patients where it correlated with tumor volumes of up to 60 cm3 | Biomarker | PD-L1 | Ricklefs et al 2018 Science Advances |
|  | EV carrying genomic cargo has a utility as a clinical biomarker | Biomarker | ATRX, IDH1, NF1, TP53, TERT | Rosa et al 2022 Biomedicines |
| Glioma | EVs from peripheral blood detect GBM mutation IDH1 G395A | Biomarker | IDH | García-Romero et al 2017 Oncotarget |
| Gastric cancer | BARHL2 methylation in gastric juice-derived exosomes may be a novel and less invasive tool for Early Gastric Cancer detection. | Biomarker | BARHL2 | Hiroyuki et al 2016 Clinical and Translational Gastroenterology |
| Gingivitis | SEC results in a higher yield of salivary sEV, with no significant differences in sEV DNA epigenetics, compared to UC | Biomarker | IL−6, TNF-α, IL−1β, IL−8, and IL−10 | Han et al 2020 International Journal of Molecular Sciences |
| Healthy pregnant women | EV DNA can be a potential source to detect fetal diseases | Diagnosis | FGFR3 | Zhang et al 2019 BioMed Central Medical Genomics |
| Hepatocellular carcinoma | EV mtDNA exhibited larger fragment size, higher copy numbers among patients with HCC and hepatitis | Biomarker | mtDNA | Li et al, 2020 Oncology Letters |
| Hepatitis B | Detection of HBV DNA in EVs when it was undetectable in plasma | Diagnosis | HBV | Sukriti et al 2018 Journal of Viral Hepatitis |
| Head and neck cancer | HPV16 DNA could be detected in isolated exosomes from the saliva of HPV-associated OPC patients | Diagnosis | HPV16 | Wang et al 2020 The Journal of Molecular Diagnostics |
| Kidney allograft injury | The majority of DNA in urine is bound to the surface of EVs and that EV DNA quantity reflects kidney allograft injury. | Biomarker | RPPH1, RPP30 | Sedej et al 2022 Journal of Extracellular Vesicles |
| Lung adenocarcinoma | ExoDNA in malignant pleural effusions could be used as a DNA source for EGFR detection in lung adenocarcinoma | Liquid biopsy | EGFR | Qu et al 2019 Frontiers in Oncology |
| Lung adenocarcinoma | Combination of High-Pure PCR Template Preparation Kit assay and targeted sequencing of EV DNA could be translated in the differential diagnosis | Liquid biopsy | ALK, ATM, APC, BRAF, CDKN2A, CHEK2, CIC, EGFR, ERBB2, EP300, KRAS, NOTCH1, PIK3CA, SMAD4, TP53, NF1, STK11, | Moa et al 2019 Analytical chemistry |
| Metastatic colorectal cancer | Exosome KRAS G12V/D can be used for liquid biopsy and provides clinical information relevant to therapeutic stratification | Liquid biopsy | KRAS | Lucchetti et al 2021 Scientific Reports |
| Melanoma | EVs derived from lymphatic drainage contains BRAF V600E mutant copies and it can be used as marker of melanoma progression. | Biomarker | BRAF | Garcia-Silva et al 2019 Journal of Experimental Medicine |
| Non-small-cell lung cancer | The combination of exosome nucleic acids and cfDNA for T790M detection has higher sensitivity and specificity compared than cfDNA alone. | Liquid biopsy | EGFR | Castellanos-Rizaldos et al 2018 Clinical cancer research |
|  | Nanoscale EV associated DNA is more sensitive than cfDNA in detecting EGFR mutations in early stage NSCLC | Liquid biopsy | EGFR, KRAS | Wan et al 2018 Annals of Oncology |
|  | EV associated DNA derived from BALF in lung cancer patients resulted in fast and accurate diagnosis. | Liquid biopsy | EGFR | Hur et al 2019 Translational Lung Cancer Research |
| Osteosarcoma | Discovery study (HSATI, HSATII, LINE1-P1, and Charlie 3 are copurified with non exosomes EVs by SEC and precipitation methods). | Biomarker | Charlie 3, HSATI, HSATII, L1P1 | Cambier et al 2021 Scientific Report |
| Ovarian cancer | EV DNA may be able to use to predict treatment response and prognosis | Liquid biopsy | ARID1A, FAT3, HNRNPA2B1, JAG2, KMT2C, MLH1, NOTCH1, NOTCH3, PTEN, TP53 | Zhao et al 2022 Cancers |
| Pancreatic ductal adenocarcinoma | Exosomes DNA outperformed ctDNA in detecting mutant KRAS in PDAC (Validation study) | Biomarker | KRAS | Allenson et al 2017 Annals of oncology |
|  | Liquid biopsy and point-of-care applications | Biomarker | KRAS, TP53 | Kahlert et al 2014 Journal of Biology Chemistry |
|  | ExoDNA is representative of the entire human genome and NOTCH1 and BRCA2, were found in patient exoDNA samples | Biomarker | BRCA2, KRAS | San Lucas et alcas 2016 Annals of Oncology |
|  | Small EVs were associated with significantly more mutant KRAS DNA | Biomarker | KRAS | Hagey et al 2021 Journal of Extracellular Vesicles |
| Parkinson Disease | The EXPAND study will provide the initial knowledge for the exploitation of EVs in Parkinson Disease management | Liquid biopsy | mtDNA | Pica et al 2019 International Journal of Molecular Science |
| Rhesus D negative pregnant women | Exosomes DNA analysis revealed higher specificity and sensitivity in RHD and SKY genotyping compared to cfDNA. However, exosome DNA is low concentration | Prenatal diagnosis and screening | GAPDH, RHD, SRY | Yaşa et al 2021 Reproductive Science |
| Sepsis | Sepsis patients have high retrotransposable elements, the study help understand of the biogenesis of cfDNAs and thus help in uncover diagnostic potential | Diagnosis | Retrotransposable elements, non-telomeric satellite DNA | Grabuschnig et al 2020 Journal of Biotechnology |
| Tuberculosis | Using ddPCR and exoDNA has the potential to provide sensitive and accurate methodology for TB diagnosis. | Diagnosis | IS6110 | Cho et al 2020 Clinical Microbiology and Infection |
| Urothelial bladder carcinoma | The study identified somatic mutation in urinary exosome DNA | Liquid biopsy | ARID1A, CCND1, CCNE1, CDKN2A, ERBB2, FGFR3, HRAS, KDM6A, KMT2D, MDM2, PIK3CA, PTEN, RB1, STAG2, TP53 | Lee et al 2018 Scientific Report |

**Supplementary table 1 b)** : EV-DNA as therapeutic applications.

| **Disease** | **Results** | **Application** | **References** |
| --- | --- | --- | --- |
| Atopic dermatitis | EV metagenomic analysis data suggested that protective role of lactic acid bacteria in Atopic Dermatitis | Therapeutic | Kim et al 2018 Allergy, Asthma and Immunology Research |
| Dermatomyositis | EVs derived from DM patients' plasma triggered proinflammatory response with STING phosphorylation. | Therapeutic | Li et al 2021 Theranostics |
| Healthy  Pregnant | Placental large EVs can Be loaded with plasmid DNA | Therapeutic | Kang et al 2023 Molecular Pharmaceutics |
| Healthy | Delivery of EV cargos as therapeutic (nucleic acids - mRNA, gDNA). | Therapeutic | Cai et al 2013 Journal Molecular cell Biology |
| Mesenchymal stem cell | MSC-EVs restore TFAM expression to prevent mtDNA damage and cytosolic mtDNA leakage in target cells and thus effectively attenuate mitochondrial damage and inflammation in cell and animal models of renal injury. | Therapeutic | Zhao et al 2021 ACS Nano |

**Supplementary table 1 c)**: EV-DNA as functional applications.

| **Disease** | **Results** | **Cargo** | Applications | References |
| --- | --- | --- | --- | --- |
| Autism spectrum disorder | EVs contain mtDNA, and they can stimulate human-cultured microglia to secrete the pro-inflammatory cytokine IL-1β. | mtDNA | Functional studies | Tsilioni et al 2018 Journal of Neuroinflammation |
| Coronary artery disease | The SRY gene in plasma EVs transferred to vascular endothelial cells may play a significant role in the pathogenesis of atherosclerosis. | SRY | Functional studies | Cai et al 2015 Clinical Science |
| Crohn disease | EV-DNA cargo aggravate Crohn's disease by activating the STING pathway | dsDNA | Inflammation | Zhao et al 2021 Cell Death Disease |
| Healthy first trimester pregnant and non-pregnant | Apoptotic bodies, nucleuses and exosomes are two major localizations of cfDNA. | EV-DNA chromatin fragment | Functional studies | Fernando et al 2018 Clinica Chimica Acta |
| Metastatic breast cancer | EVs play a role in therapy resistant and metastasis. | dsDNA | Oncogenesis | Sansone et al 2017 Proceedings of the National Academy of Sciences of the United States of America |
